# [Retracted] DNA methyltransferase 3b silencing affects locus‑specific DNA methylation and inhibits proliferation, migration and invasion in human hepatocellular carcinoma SMMC‑7721 and BEL‑7402 cells

**DOI:** 10.3892/ol.2024.14819

**Published:** 2024-11-25

**Authors:** Jia-Chen Wang, Zhao Wang, Yu-Xia Fan, Ya-Qing Si, Jia-Xiang Wang

Oncol Lett 9: 2499–2506, 2015; DOI: 10.3892/ol.2015.3077

Following the publication of the above paper, it was drawn to the Editor's attention by a concerned reader that Fig. 3A and C on p. 2504 contained a pair of duplicated data panels, such that data which were intended to show the results from differently performed experiments appeared to have been derived from the same original source. Following an independent investigation of this matter in the Editorial Office, it came to light that certain of the aforementioned data in Fig. 3A and C had already been published in an article in the journal *PLoS Genetics* (doi.org/10.1371/journal.pgen.1000879) written by different authors at different research institutes. Owing to the fact that the contentious data in the above article had already been published prior to its submission to *Oncology Letters*, the Editor has decided that this paper should be retracted from the Journal. The authors were asked for an explanation to account for these concerns, but the Editorial Office did not receive a reply. The Editor apologizes to the readership for any inconvenience caused.

